# Re-examining content validity of the BREAST-Q more than a decade later to determine relevance and comprehensiveness

**DOI:** 10.1186/s41687-023-00558-y

**Published:** 2023-04-06

**Authors:** Manraj N. Kaur, Sabrina Chan, Louise Bordeleau, Toni Zhong, Elena Tsangaris, Andrea L. Pusic, Stefan J. Cano, Anne F. Klassen

**Affiliations:** 1 Brigham and Women’s Hospital, Harvard Medical School, 75 Francis S, Boston, MA 02115 USA; 2 McMaster University, 1280 Main Street W, Hamilton, ON L8S 4L8 Canada; 3 Juravinski Cancer Center, Room 3-17, 699 Concession Street, Hamilton, ON L8V 5C2 Canada; 4 Toronto General Hospital, 8N-871, Norman Urquhart Wing, Toronto, ON M5G 2C4 Canada; 5 Modus Outcomes, 4th Floor St. James House, St. James Square, Cheltenham, England, GL50 3PR UK

**Keywords:** Breast cancer, Qualitative, Content validity, BREAST-Q, Lymphedema, Breast sensation, Comprehensiveness, Cancer worry, Fatigue, Work impact, Patient-reported outcome measures, Patient-reported outcome

## Abstract

**Purpose:**

The BREAST-Q is the most used patient-reported outcome measure (PROM) in breast cancer surgery. The purposes of this study were to re-examine the content validity of BREAST-Q cancer modules (mastectomy, lumpectomy and reconstruction) and to determine the need for new scales.

**Methods:**

Interviews were conducted with women with breast cancer (Stage 0–4, any treatment), and were audio-recorded and transcribed verbatim. Deductive (based on original BREAST-Q conceptual framework) and inductive (new codes from the data) content analysis approaches were used to analyze the data. The number of codes that mapped to BREAST-Q were recorded.

**Results:**

Dataset included 3948 codes from 58 participants. Most of the breast (*n = *659, 96%) and all psychosocial (*n = *127, 100%), sexual (*n = *179, 100%) and radiation-related (*n = *79, 100%) codes mapped to BREAST-Q Satisfaction with Breast, Psychosocial Wellbeing, Sexual Wellbeing and Adverse Effects of Radiation scales, respectively. For the physical wellbeing codes (*n = *939) for breast/chest and arm, 34% (*n = *321) mapped to the Physical Wellbeing-Chest scale. Most of the abdomen codes (*n = *311) mapped to Satisfaction with Abdomen (*n = *90, 76%) and Physical Wellbeing-Abdomen (*n = *171, 89%) scales. Codes that did not map (*n = *697, 30%) covered breast sensation and lymphedema. Concerns related to fatigue, cancer worry, and work impact were most reported and did not map to BREAST-Q.

**Conclusion:**

The BREAST-Q, which was developed using extensive patient input more than a decade ago, is still relevant. To ensure the BREAST-Q remains comprehensive, new scales for upper extremity lymphedema, breast sensation, fatigue, cancer worry, and work impact were developed.

## Background

The United States Food and Drug Administration (US FDA) Guidance for Industry Patient-Reported Outcome Measures [1], the COnsensus-based Standards for the selection of health Measurement INstruments (COSMIN) [2, 3], the International Professional Society for Health Economics and Outcomes Research (ISPOR) [4, 5], the Medical Outcomes Trust [6, 7] and various articles published in the health-related quality of life (HRQL) measurement literature address the importance of demonstrating the content validity of a patient-reported outcome measure (PROM) before other measurement properties of reliability, validity, and responsiveness are evaluated. Content validity is the extent to which a PROM is relevant to, and representative of the targeted construct it is designed to measure [1]. Content validity is optimally established with the qualitative studies (one-on-one interviews or focus groups) that are conducted to demonstrate that the individual items and the instrument as a whole is relevant, comprehensive and comprehensible relative to the construct it intends to measure and in the target population, health condition, treatment, or context of use[1, 2]. Without content validity, evidence of other measurement properties, such as reliability and other types of validity (e.g., construct, criterion), is essentially meaningless. As such, content validity is the most important psychometric property of an instrument, and a yardstick to gauge if the PROM is well developed.

**Fig. 1:**
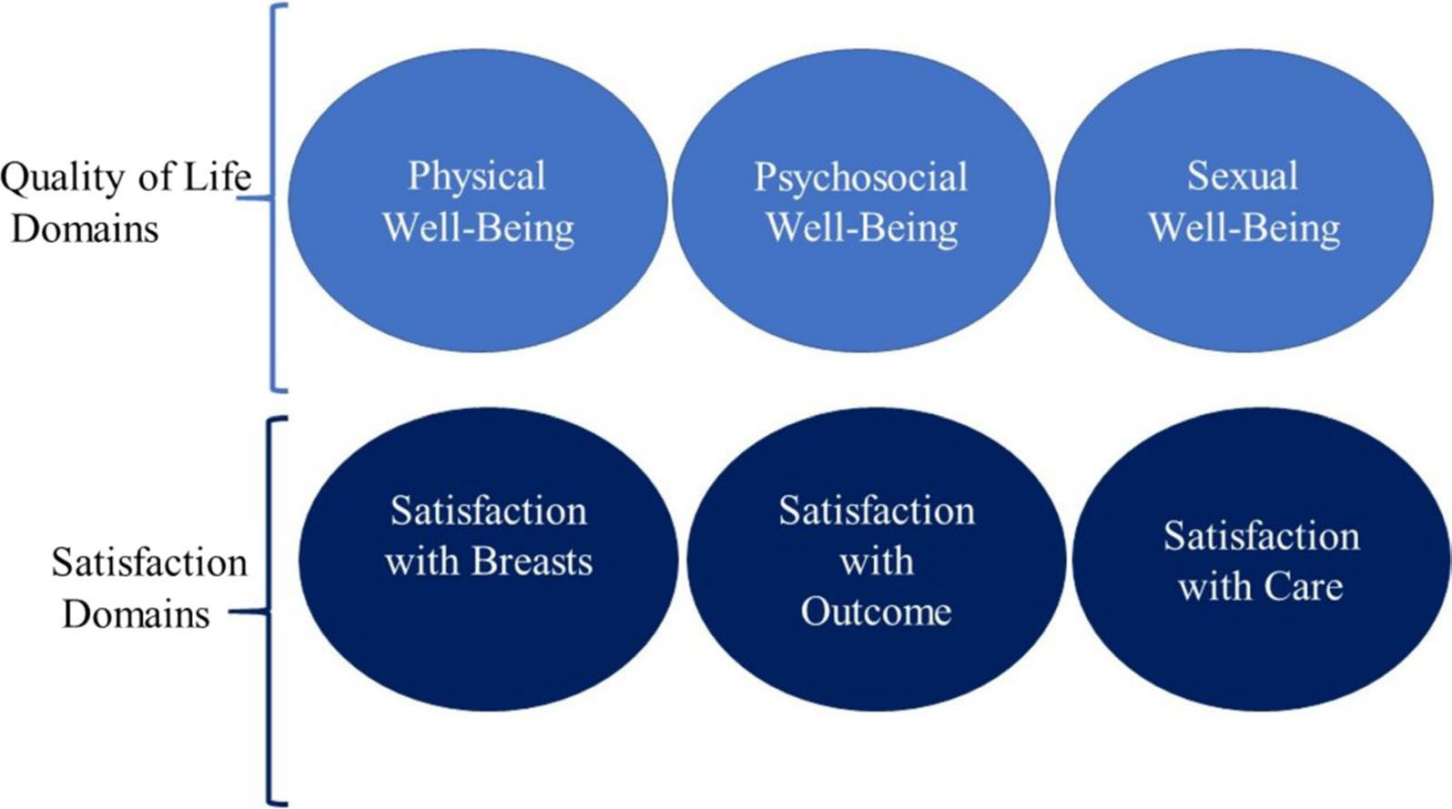
Original conceptual framework of BREAST-Q [8] (Reproduced with permission)

The BREAST-Q [8] is a modular PROM for breast surgery published in 2009, in accordance with the standards and guidelines drawn from the literature available at that time. To develop the BREAST-Q, in 2004, in-depth qualitative interviews were conducted with 48 women who were seeking or had undergone breast surgery. Data were analysed and used to develop a conceptual framework and preliminary BREAST-Q scales. These scales were shown to clinician experts who were invited to nominate missing items [8, 9]. The conceptual framework and BREAST-Q scales were refined and shown to 58 women who took part in two separate focus groups. Further feedback from clinical experts was sought. These sessions were used to establish relevance and comprehensiveness of the conceptual framework and the preliminary BREAST-Q scales. Final refinements were made to the BREAST-Q based on cognitive debriefing interviews with 30 women who provided feedback on the relevance, comprehension, and comprehensibility of the BREAST-Q items. The content validity of the BREAST-Q was, thus, well supported by extensive evidence from qualitative studies. The original conceptual framework of the BREAST-Q is shown in Fig. 1. The BREAST-Q was field-tested in a sample of 1950 women and Rasch Measurement Theory analysis was used to establish psychometric properties. The BREAST-Q has three modules for breast surgery – mastectomy, breast conserving therapy (BCT) and reconstruction [8–10].

Since its development, the BREAST-Q has been used globally to reliably and meaningfully measure HRQL and patient satisfaction in clinical practice, research, and quality improvement initiatives in women with breast cancer surgery [11, 12]. However, clinical evidence on breast cancer- and breast cancer surgery-related intervention evolves rapidly. As the understanding of related health conditions (e.g., breast cancer-related lymphedema) grows and new treatments become available, the preferences and values of women seeking breast cancer surgery evolve in parallel. The discourse of women with breast cancer used to describe what matters to them also changes. It is of utmost importance to ensure that high-uptake PROMs, i.e., the BREAST-Q, remain comprehensive, relevant, and that there are no gaps in the constructs being measured. Hence, the purpose of this study was to re-examine the content validity of the BREAST-Q cancer modules (i.e., breast conserving surgery, mastectomy, and breast reconstruction) a decade after its development.

## Methods

To re-examine content validity of the BREAST-Q, a secondary analysis of an existing qualitative dataset collected for  a program of research to develop the BREAST-Q Utility module was conducted [13]. The protocol and the results  for the development of the Utility module are published elsewhere [13, 14]. Briefly, as part of the BREAST-Q Utility module development, a purposive sample of English-speaking, adult women (18 years and older) diagnosed with breast cancer (any stage, any treatment) was recruited from three tertiary healthcare centers – two in Ontario, Canada (Juravinski Cancer Center, Hamilton and Toronto General Hospital, Toronto) and one in the United States (Memorial Sloan Kettering Cancer Center, New York). The sample varied by age, stage of breast cancer, and type of treatment. Once written informed consent was obtained, in-depth, semi-structured interviews were conducted in-person or over the telephone. An interview guide was used [see 13] and refined iteratively throughout the study to add new concepts and probes. The interviews were audio-recorded, transcribed verbatim and analysed line-by-line. Content analysis of the data was completed using an approach that involved the application of deductive (codes mapped to the BREAST-Q framework) and inductive (new codes that emerged from the data) codes. Constant comparison was used to organize the codes into top-level domains, subdomains and major and minor themes. Interviews continued until redundancy was thought to be achieved at the level of minor themes for the development of the BREAST-Q Utility module.

Subsequently, a secondary analysis was conducted whereby two independent qualitative researchers, examined each code used to develop an item in the item pool and mapped these to the BREAST-Q item that most closely reflected the concept of interest. For each concept that was mapped, the corresponding BREAST- Q item and scale was recorded. In the BREAST-Q, the Psychosocial and Sexual Wellbeing scales, and the Adverse Effects of Radiation scale are the same across the three breast surgery modules. The Physical Wellbeing scale is also the same in the three modules, except for 2 items - pain in the muscles in the chest and nagging pain - that are not included in the BCT module. Similarly, there is an overlap in the items in the Satisfaction with Breasts scale in the mastectomy module and the BCT and reconstruction module, with the exception of one item in the BCT Satisfaction with Breasts scale that asks about how smoothly shaped the breasts are. Consequently, the content mapping was performed at the scale-item level and not at the module-scale-item level.

For items about breast scars that did not map to the BREAST-Q, the SCAR-Q [15] scales were reviewed for relevance and comprehensiveness. We mapped to the SCAR-Q because this PROM included all the scar-specific codes from BREAST-Q in its development [15].

Codes pertaining to systemic cancer treatment (chemotherapy, endocrine therapy, or targeted therapy) or cancer diagnosis were recorded as “not applicable” as the original BREAST-Q conceptual framework did not include these concepts. The frequency of the codes that mapped to the BREAST-Q items was calculated and used to identify gaps in the BREAST-Q. Regular meetings were held with the research team to ensure rigour in the analysis.

## Results

The qualitative dataset included 3948 codes from 58 women aged 55 ± 10 years (range 22–75 years). The participant sample was diverse with respect to the stage of breast cancer (stages 0–4; 76% with stage 0–2), racial and ethnic background (79% White), marital status (76% married/living common law), employment status (43% employed full-time), the highest level of education completed (60% College/University or higher), and total household income from previous year (53% over USD/CAD 75,000). Table 1 shows detailed demographic and clinical characteristics of the study population. Table 2 shows the frequency of codes that mapped to the BREAST-Q existing scales by the body part and domain of HRQL.

**Table 1:** Demographic and clinical characteristics of the study population (*n = *58)

*N = *58	*N*	%
*Age in years*		
Young adult (18–39)	2	3.5
Middle-aged adult (40–59)	40	68.9
Older adult (60 and above)	16	27.6
*Race/Ethnicity*		
Caucasian	46	79.3
Black or African American	2	3.5
Asian	5	8.6
Other	5	8.6
*BMI category*		
Underweight – 18.49 and lower	2	4.1
Normal – 18.5 to 24.9	19	38.8
Overweight – 25 to 29.9	20	40.8
Obese – 30 and higher	8	16.3
*Marital status*		
Married/Living common law	44	75.9
Single, never married	4	6.9
Divorced/Separated/Widowed	10	17.2
*Employment*		
Employed, full-time	25	43.1
Employed, part-time	12	20.7
Unemployed	2	3.5
Homemaker	3	5.2
Sick leave/Disabled	3	5.2
Retired	11	18.9
Other	2	3.5
*Total annual household income (previous year)*		
0–25,000	5	8.6
25,000–50,000	5	8.6
50,000–75,000	8	13.8
> 75,000	31	53.5
Prefer not to say	9	15.5
*Education*		
High school graduate or equivalent	10	17.2
Some college/University (less than 4 years)	13	22.4
College/University (4-year Bachelor’s degree)	28	48.3
Postgraduate degree (e.g., Masters, Doctorate, etc.)	7	12.1
*Type of (neo)adjuvant treatment*		
Chemotherapy	38	65.5
Radiation	36	62.1
Hormone replacement therapy	37	63.8
Targeted therapy (HER2)	7	12.1
*Type of cancer surgery*		
Breast conserving therapy	9	15.5
Mastectomy – unilateral	25	43.1
Mastectomy – bilateral	23	39.7
None	1	1.7
*Reconstruction*	*N = 48*	
Yes	36	
No	12	
*Type of reconstruction*	*N = 36*	
Autologous	26	75
Implant	10	25
*Laterality*		
Unilateral	18	50
Bilateral	18	50
*Timing of reconstruction*		
Immediate	21	58.3
Delayed	6	16.7
Not available	9	25

**Table 2:** Percentage of codes that mapped to the BREAST-Q scales

Scale	Total number of codes	Number of codes that mapped (%)
Satisfaction – Breast (including Nipple, Cleavage, Scar, Implant)	659	630 (95.6%)
Physical Wellbeing – Breast (including Breast Sensation and Arm Lymphedema)	939	321 (34.2%)
Adverse Effects of Radiation	79	79 (100%)
Psychosocial Wellbeing	127	127 (100%)
Sexual Wellbeing	179	179 (100%)
Satisfaction – Abdomen	119	90 (75.6%)
Physical Wellbeing – Abdomen	192	171 (89.1%)
Cancer-related (including Social Relationships)	1654	

Table 3 shows the mapping of the qualitative codes to the BREAST-Q items, by concepts. For the psychological distress or distress-related impact (127 codes), sexual (179 codes), and adverse effects of radiation (79 codes), the qualitative codes mapped fully to the respective BREAST-Q scales. A total of 939 codes were about the physical wellbeing of the chest for the breast area and arm (i.e., symptoms and function). The codes that did not map included 230 codes about breast sensation and 14 codes about swelling of the breast post-breast cancer surgery. Further, a total of 399 codes were about arm swelling (i.e., upper extremity lymphedema) post-breast cancer surgery, out of which 25 were about swelling of the arm and 90 were about difficulty with lifting or moving the arm. The codes about arm swelling and difficulty with arm movement mapped to the respective BREAST-Q Physical Wellbeing scale item. However, apart from arm swelling and mobility, women also described the appearance of the arm affected by lymphedema, and other lymphedema symptoms such as arm sensation, pain, fatigue and changes in dexterity and grip strength. In addition, women with arm lymphedema talked about the impact of lymphedema on their sleep, work, and daily activities, and the impact of wearing a compression sleeve on clothing choices and body image.

**Table 3:** Mapping of codes to the BREAST-Q scales

Domain	BREAST-Q Item	No. of codes that mapped per item	Subcodes (where applicable)
Satisfaction-breast(s) (*n* = 485)	Look in the mirror-clothed	39	
	Shape of breast(s)-bra on	26*	
	Feel normal-clothed	8	
	Size of breast(s)	19	
	Wear fitted clothing	55*	
	Breasts lined up	20	
	Bra fit	31	
	Softness of breast(s)	45	
	Breasts equal in size	26	
	Breast(s)-natural looking	16	
	Breast(s)-sit/hang	11	
	Breasts-touch	25	
	Breasts-part of body	23	
	Breasts-matched	34	
	Look in mirror-clothed	74*	
	Implant-Rippling/Wrinkling	5	
	*Clothing type*	*24*	
	*Movement*	*4*	
Satisfaction—nipples and areola (*n* = 44)	Size	5	
	Shape	2	
	Natural	4	
	Color	11	
	Projection	7	
	Position	1	
	Similar	1	
	Sensation	5	
	*Soft*	*1*	
	Look overall	7	
Satisfaction – cleavage (*n* = 14)	Scale under development		
Satisfaction – scar (*n* = 116)	Mapped to SCAR-Q		
Psychological wellbeing (*n* = 127)	Confident-social situation	24	
	Emotionally able	1	
	Emotionally healthy	21	
	Equal worth to other women	1	
	Self-confident	4	
	Feminine	1	
	Accepting of body	50	
	Normal	3	
	Like other women	15	
	Attractive	7	
Physical wellbeing – chest (*n* = 565)	Pain-chest	69	
	Difficulty moving/lifting arm	58	
	Difficulty sleeping	25	
	Tightness-breast area	61	
	Pulling-breast area	42	
	Nagging feeling	1	
	Tenderness	1	
	Sharp pain	24	
	Aching feeling	8	
	Throbbing	7	
	Arm swelling	25	
	*Other Arm codes^*	*374*	
	*Breast swelling*	*14*	
	*Breast sensation*	*230*	*Numbness (n* = *71)* *Tingling (n* = *34)* *Heavy (n* = *27)* *Temperature changes (n* = *19)* *Itchy (n* = *12)* *Pressure (n* = *9)* *Other abnormal sensation (n* = *58) eg, burning, milk let-down, twinges*
	*Comfortable/at ease*	*44*	*At ease-overall (n* = *8)* *At ease re. partner touching/looking at breasts (n* = *34)* *At ease re. touching own breasts (n* = *2)*
	Confident	8	Self-conscious (*n* = 2) Confident (*n* = 6)
	Satisfied	55	Drive/arousal (*n* = 11) Frequency (*n* = 33) Quality (*n* = 7) Overall (*n* = 4)
	Confident-breast appearance	23	Appearance of breasts and nipple (*n* = 23)
	Sexually attractive-unclothed	30	Look overall-unclothed (*n* = 30)
Adverse effects of radiation (*n* = 79)	Skin-look different	40	Color changes (*n* = 5) Overall different (*n* = 35)
	Marks on skin	3	Brownish-black marks/looks burnt (*n* = 3)
	Skin-dry	7	Dry (*n* = 3) Itchy (*n* = 4)
	Skin-sore to touch	17	Sore/painful/discomfort/tender (*n* = 9) Sensitive (*n* = 8)
	Skin-thicker when touched	11	Feels different (*n* = 11)
	Skin-irritated by clothing	1	Clothing rubs against skin (*n* = 1)
Other arm codes (*n* = 374)	*Physical wellbeing*	*135*	*Function* *Activity avoidance/behaviour (n* = *27)* *Work impact (n* = *11)* *Difficulty sleeping (n* = *7)* *Difficulty lifting or moving arms (n* = *90)*^*#*^
		*103*	*Symptoms* *Sensation-arm (n* = *39)* *Pain (n* = *33)* *Fatigue (n* = *17)* *Skin changes (n* = *6)* *Dexterity/Grip (n* = *6)* *Pulling (n* = *2)*
	*Psychological wellbeing*	*29*	*Distress*
	*Satisfaction*	*47*	*Arm sleeve*
		*60*	*Arm appearance*

Codes highlighted in italic did not map to the BREAST-Q; ^arm codes that pertained to lymphedema are shown separately; #mapped to BREAST-Q Physical Wellbeing difficulty moving or lifting arm item

For Satisfaction with Breasts, the qualitative dataset included 659 codes that were about the breasts overall, nipples, cleavage and breast scar (Table 3). A majority (96%) of the codes mapped to the BREAST-Q scale. See Appendix for an example of patient quotes that mapped to the BREAST-Q Satisfaction with Breasts scale. The codes that did not map covered the type of clothing women were able to wear post-breast cancer surgery, and if and how much the breasts moved post-breast reconstruction. Codes about how the nipples felt post-surgery (e.g., soft) did not map to the Nipple scale. The codes about breast scars fully mapped to the SCAR-Q Appearance scale.

Out of the 58 women in the study sample, 26 women underwent abdomen-based reconstruction. The abdomen-related codes (311 codes, Table 4) included codes about physical wellbeing and satisfaction with the appearance of the abdomen (including belly button and abdomen scar[s]). While most codes mapped to the BREAST-Q Physical Wellbeing-Abdomen and Satisfaction with Abdomen scales, the codes that did not map pertained to accommodations in the immediate postoperative period (e.g., belly brace, shower chair or hospital bed) and lack of or abnormal sensations in the abdomen area, along the abdominal scar or around the belly button. Additionally, 29 codes were about how the abdomen looked in fitted clothing or the ability to wear fitted clothing post-abdomen-based breast reconstruction. These codes did not map to the current BREAST-Q Satisfaction with Abdomen scale.

**Table 4:** Mapping of codes to the BREAST-Q abdomen scales (for abdomen-based reconstruction)

Domain	BREAST-Q Item	No. of codes that mapped per item	Subcodes where applicable
	Look – unclothed	18	
Satisfaction – abdomen (*n* = 119)	Position-belly button	11	
	Appearance-scars	61	Overall (*n* = 31) Location (*n* = 19) Shape (*n* = 10) Size (*n* = 1)
	Fitted clothing	29	Scar (*n* = 15) Abdomen (*n* = 12) Belly button (*n* = 2)
Physical wellbeing – abdomen (*n* = 192)	Sitting up	15	
	Everyday activities	27	ADL (*n* = 15) Lifting object (*n* = 5) Move around (*n* = 4) Walk (n = 3)
	Discomfort	64	ADL (*n* = 53) Sleep (*n* = 7) Cough (*n* = 3) Touch (*n* = 1)
	Bloating	9	
	Bulging	7	
	Tightness	32	
	Pulling	17	
	Sensation	16	Abdomen (*n* = 9) Scar (*n* = 6) Around the belly button (*n* = 1)
	Accommodations	5	Belly brace, shower chair, recliner

Codes highlighted in italic did not map to the BREAST-Q;ADL, activities of daily living

A total of 1654 codes were specific to systemic breast cancer treatments (i.e., chemotherapy, endocrine therapy and targeted therapy). Women described the issues pertaining to symptoms, function, psychological distress, and social participation (i.e., work impact, isolation, social support and relationships). The three most common themes described by women pertained to the experience and impact of fatigue, cancer worry and work. The cancer-related codes did not map to the BREAST-Q.

## Discussion

The purpose of the study was to re-examine the content validity of the BREAST-Q more than a decade after its development using secondary qualitative data analysis. We found that while most of the qualitative codes from this recent set of interviews with a heterogeneous sample of women with breast cancer mapped to the items forming the BREAST-Q cancer surgery module scales, some key concepts were missing. These concepts included HRQL and appearance-related issues pertaining to upper extremity lymphedema, breast sensation, and cancer-specific concerns (i.e., fatigue, cancer worry, and work impact).

The content of the BREAST-Q Satisfaction with Breast scale, Psychosocial and Sexual Wellbeing, Adverse Effects of Radiation, Satisfaction with Abdomen and Physical Wellbeing-Abdomen was found to be comprehensive and relevant to women with breast cancer. For codes related to breast scars, the SCAR-Q-Appearance scale was found to be comprehensive, which is understandable as the scar codes from the original qualitative BREAST-Q development dataset were used to inform the development of the SCAR-Q [15]. Subsequently, clinicians and researchers interested in measuring scars (appearance, symptoms or psychosocial impact) following breast cancer surgery, should consider using the SCAR-Q. This study demonstrated that when rich, in-depth qualitative input from patients is sought to develop PROMs and demonstrate their content validity, the PROM remains relevant and comprehensive for many years.

The notion of (re)examining content validity of PROMs in an iterative manner years after the development of the PROM has rarely been documented in the literature. However, in the educational assessment literature – from where most psychometric methods have evolved – it is common practice to evaluate content of examination tests periodically to ensure they reflect the current knowledge and individual-level and societal factors [16–19]. Doing so, lays the foundation to substantiate the content validity of the tests, and subsequently the entire examination process. This lack of prospective content validation of PROMs in health measurement literature is an important “transfer of knowledge gap” from the educational literature.

The lack of prospective content validation of PROMs is particularly relevant in the context of breast cancer. The rapid increase in evidence on the pathophysiology of breast cancer and related conditions (e.g., arm lymphedema) in the last decade has resulted in new, targeted, and patient-centered treatment interventions that are quickly made available to the patient population. Further, with the advancements in evidence-based breast cancer surgery, patient goals that were previously considered farfetched may soon become reality. The evolution of microsurgical techniques to reestablish breast sensation following breast reconstruction in the last 5 years is a fitting example. While the evidence on the ideal approach to reinnervation of breasts continues to evolve, the variability in how breast sensation is measured and the lack of inclusion of PROMs have been noted [20]. Our program of research identified breast sensation as a relevant concept to patients that was not included in the BREAST-Q. To address this gap, our team  has developed and validated the BREAST-Q Sensation Module that consists of three independently functioning scales measuring breast symptoms, sensation and quality of life impact of sensation loss [21]. We also developed new BREAST-Q scales for the three most commonly reported breast cancer concepts - fatigue, cancer worry and work impact [22]. Similarly, for upper extremity lymphedema, a new PROM called the LYMPH-Q Upper Extremity was developed to measure arm function, symptoms, arm appearance, psychological impact, lymphedema information and arm sleeve satisfaction [23]. We conducted additional interviews with women with breast cancer-related lymphedema (n = 12) to address concepts that were identified as important in this qualitative dataset but did not reach theoretical saturation. Consequently, by closely re-examining the content validity of the BREAST-Q, we were able to identify gaps in PRO measurement in breast cancer surgery and add new scales to the BREAST-Q and develop the LYMPH-Q Upper Extremity module.

Notably, a topic of contention within the content validity literature is how often and in what circumstances the content validity of a PROM should be assessed. The US FDA Guidance document, the COSMIN and the ISPOR guidelines recommend that if the intended use of the PROM is changed with respect to the target population, health condition or treatment, context of use and the construct being measured, the content validity of the instrument should be re-assessed. This ensures that the items and domains of the PROM are “fit for purpose” and relevant to the target population. However, a working meeting of the Patient Reported Outcomes Measurement Information System (PROMIS) initiative on the topic of content validity expressed concerns over this guidance [24]. At this meeting, it was argued that the need to re-certify the content validity of a rigorously developed and established PROM each time it is used in a new clinical group may slow down the clinical and research-related implementation of a PROM. The group recommended that instead of establishing content validity every time, measures of common symptoms or function could be considered “reasonable for use”, while examining generalizability. Furthermore, the group suggested that rather than asking if an instrument is valid, one should ask what makes the instrument invalid in the specific context [24]. Our study challenges both guidances and demonstrates that even if the context of use and the intended population remain the same, for health conditions (and subsequently for condition-specific PROMs), where substantial innovation and changes in treatment protocols and guidelines occur, establishing prospective content validity is a worthwhile exercise.

Our study has certain limitations that are due to the use of secondary data analysis and the difference in framing of the BREAST-Q items and the qualitative codes. As the original purpose of the study was not to re-examine content validity of the BREAST-Q, it is likely that there may be additional missing concepts that were not identified. However, our analysis demonstrated that BREAST-Q has retained its content validity a decade later, and hence, we hypothesize that a study designed to specifically re-establish content validity of the BREAST-Q will only strengthen our conclusion. Further, none of the patients in the qualitative study had had Latissimus Dorsi-based breast reconstruction so the content validity of scales relevant to that procedure could not be established. Another limitation is that some scales in the BREAST-Q are framed positively. This is particularly relevant for the Psychosocial Wellbeing scale, whereby the items ask about “positive affect” and “positive body image” rather than distress. For example, instead of asking about anxiety or depression and social isolation, the scale asks about feeling emotionally healthy, emotionally able to do things that one would like to do, and feeling confident in social settings, respectively. In the qualitative dataset, women often used negative terms to describe their psychosocial wellbeing, especially early in their cancer trajectory. To overcome this, the data were coded by two independent coders who carefully considered the psychosocial codes and only codes that logically and intuitively mapped to the BREAST-Q items were mapped as “yes”. Current research in survey methodology shows that wording questions positively or negatively systematically affects the answers [25, 26], and positively worded items have been found to be more helpful in measuring more severe manifestations of a construct [27]. Hence, future studies should use qualitative evidence to explore the cognitive concurrence of the positive psychological items and negative words used by the participants for the BREAST-Q Psychological Wellbeing scale, and other positively worded PROMs.

In conclusion, the breast cancer surgery modules of the BREAST-Q demonstrated content validity more than a decade after its development. The modular structure of the BREAST-Q is the key feature that allows for addition of new concepts and modules as they are identified in clinical practice and research. When the development of a PROM is done right, it impacts evidence-based practice at multiple levels – from treatment-decision making, evaluation of treatment outcomes, and to treatment reimbursement. Hence, as treatments evolve, the HRQL impact of the treatments, patient preferences, and the language used by the patients to describe their experiences evolve in parallel. Subsequently, the content of a PROMs needs to be evaluated on an ongoing basis to ensure that it remains “fit for the purpose”. The development and validation of PROMs is resource and time-consuming task, but it should not be treated as the ultimate endpoint.

## Data Availability

The datasets used and/or analysed during the current study are available from the corresponding author on reasonable request.

## Appendix

See Table 5.

**Table 5:** Sample patient quotes from the qualitative dataset for the BREAST-Q Satisfaction with Breast scale

BREAST-Q item*	Sample Quote from the interviews
In the past week, how *satisfied or dissatisfied* have you been with…	
a. …look in the mirror-clothed	“…My breasts look normal with clothes on…” (61–3-Ab)
b. …shape of breast(s) – bra on	“…My reconstructed breast looks the same as the other breast with bra on…” (63–2-Ab)
c. …feel normal – clothed	“…I look normal with clothes…” (53–1-Ab)
d. … size of breast(s)	“…I am happy with the size of my breasts…” (58–0-Im)
e. …wear fitted clothing	“…I am able to wear more fitted clothes…” (57–2-Ab)
f. …breasts lined up	“…My breasts are not in line with each other…”(63–2-Ab)
g. … bras fit	“…My bra doesn’t fit perfectly…” (40–3-Ab)
h. …softness of breasts	“…My reconstructed breasts are soft…” (62–0-Ab)
i. …breasts equal in size	“…My breast are equal in size…” (63–2-Ab)
j. … breast(s) look natural	“…My breasts look natural when I have bra on..” (63–2-Ab)
k. …breast(s) sits/hangs	“…The right breast doesn’t hang naturally…” (63–2-Ab)
l. …breast(s) feels to touch	“…Touching my breast bothers me…” (58–0-Im)
m. …breast(s) feels like part of body	“…It's a part of my body now…” (57–0-Ab)
n. …breasts closely matched	“…The breasts are not perfect match …” (42–1-Im)
o. ……look in the mirror-unclothed	“…I don’t like what I see when I look at my breast area in the mirror without clothes on…” (40–3-Im)

^*^BREAST-Q items have been abbreviated. For a review copy of the BREAST-Q Satisfaction with Breasts scale, please visit www.qportfolio.org

Participant – Age-Stage of Breast Cancer-Type of Reconstruction, Ab- Abdomen based, Im-Implant
